# Generative Design of 3D-Printed Biomimetic Interlocking Blocks Inspired by the Cellular 3D Puzzle Structure of the Walnut Shell

**DOI:** 10.3390/biomimetics11040289

**Published:** 2026-04-21

**Authors:** Alexandros Efstathiadis, Ioanna Symeonidou, Konstantinos Tsongas, Emmanouil K. Tzimtzimis, Dimitrios Tzetzis

**Affiliations:** 1 Digital Manufacturing and Materials Characterization Laboratory, School of Science and Technology, International Hellenic University, 57001 Thermi, Greece; 2 Department of Architecture, University of Thessaly, 38221 Volos, Greece; 3 Department of Industrial Engineering and Management, School of Engineering, International Hellenic University, 57001 Thermi, Greece

**Keywords:** biomimicry, walnut shell, generative design, 3D printing, three-point bending, finite element analysis

## Abstract

The goal of the present paper is to apply a novel biomimetic design strategy for the analysis, emulation, and technical evaluation of design solutions inspired by the morphogenetic logic of the walnut shell microstructure. The shell consists of specialized cells, called sclereids, which develop protrusions and mechanically interlock with neighboring cells, providing exceptional toughness through increased surface contact. To extract and transfer this biological principle, a generative algorithm was developed using the evolutionary solver Galapagos within the Grasshopper visual programming environment. The algorithm generates protrusions on the interfaces of structural blocks and optimizes their contact surface area while maintaining constant block volume. Additional design constraints, including symmetry and manufacturability considerations, were introduced to improve structural performance and computational efficiency. A series of physical specimens with variations in key geometric parameters, such as protrusion number and height, were fabricated using fused filament fabrication (FFF) with PLA material and evaluated through in-plane and out-of-plane three-point bending tests. The results show that increasing the number of protrusions significantly enhances mechanical performance, while increasing their height improves stiffness and interlocking up to a certain threshold, beyond which structural performance decreases due to stress concentration effects. This behavior can be attributed to improved load transfer and stress distribution across the enlarged interfacial area, as well as progressive mechanical engagement between complementary protrusions. The computational model is in good agreement with the experimental results, confirming the validity of the proposed approach. The study demonstrates that biomimetic optimization of interfacial geometry can enhance the mechanical behavior of interlocking systems and provides a framework for translating biological morphogenetic principles into engineering design applications.

## 1. Introduction

Nature is governed by mathematical patterns and constructional logic that determine how biological structures are formed [1,2]. By examining these natural systems and understanding their morphogenetic rules, it is possible to derive design algorithms that describe the mechanisms of their shape and growth [1,3,4]. Modern computational design approaches such as parametric, generative, and algorithmic design can be used to extract and transfer these rules, enabling the development of biomimetic designs informed by the underlying principles that determine biological form and function, a process known as design by analogy [5,6,7,8]. These approaches generate forms and patterns through rule-based and algorithmic processes that approximate morphogenetic phenomena [9,10]. Ultimately, computational design enables the development of complex biomimetic solutions that would be difficult to achieve using conventional techniques, with applications in architecture, design, and engineering [11,12,13].

More specifically, Fischer and Herr defined generative design as an approach in which the designer interacts indirectly with the model through a generative system, where the virtual design space is explored in a manner analogous to evolutionary processes [14,15]. These systems enable the exploration of a wide design space, more or less autonomously, identifying solutions that satisfy specific performance criteria and constraints [16,17,18]. Generative design allows the creation and exploration of complex forms and patterns, as each new combination of parameters enables the emergence of new design solutions and topologies [11,19,20].

Additive manufacturing, commonly known as 3D printing, simplifies the fabrication of complex geometries by producing objects layer by layer from digital models [21,22,23]. It enables the production of high-precision organic structures without the limitations of conventional subtractive manufacturing methods [23,24,25]. These characteristics make it particularly suitable for the fabrication and prototyping of biomimetic structures [26,27,28].

Biological shells are complex hierarchical structures that exhibit exceptional morphological and mechanical characteristics [29,30]. Nuts are dry, one-seed fruits with a hardened shell (endocarp) that protects the seed from environmental threats [31,32,33]. These shells demonstrate high strength and toughness due to complex hierarchical arrangements that combine organic and inorganic components into structures with remarkable fracture resistance and stability [34,35,36,37,38,39,40]. Such systems achieve high mechanical performance despite being composed of relatively weak base materials [41,42].

Recent research on interlocking block and brick systems highlights their potential to improve structural efficiency, seismic resilience, thermal performance, and construction sustainability across a wide range of materials and geometries. Studies on mortar-free concrete interlocking blocks demonstrate how mechanical interlock, frictional interfaces, and energy-dissipating mechanisms enable stable load transfer and enhanced earthquake performance [43,44]. Other work on 3D-printed modular geometries, including PolyBrick ceramic units and other bio-inspired assemblies, shows how digitally fabricated interlocks allow for precise load paths, complex curvature, and customizable mechanical behavior while reducing reliance on mortar and enabling reversible construction [45,46]. Experimental studies on compressed-earth and aerogel-based interlocking bricks further highlight how optimized interlock morphology can increase compressive capacity, improve insulation, and simplify low-cost housing construction [47,48]. Advantages of such systems include ease of assembly, reduced need for skilled labor, reduced construction costs, design flexibility, and rapid deconstruction. However, challenges such as long-term performance and lack of standardized testing frameworks remain [43,45].

Despite the extensive research on interlocking structural systems, most existing approaches rely on predefined geometries and empirical design rules, with limited exploration of systematically optimized interface morphologies [43,44,45,46,47,48,49]. At the same time, biomimetic design studies often focus on formal resemblance rather than the direct translation of underlying biological mechanisms into engineering parameters [26,27,28,50]. In particular, the role of surface-driven interlocking, as observed in biological systems such as the walnut shell, remains largely underexplored in engineered interlocking structures [49,51,52]. Furthermore, although generative and evolutionary design methods have been widely applied in architectural and engineering contexts, their integration with biomimetic principles and experimental validation remains limited [53,54]. Existing studies rarely establish a direct link between biological morphogenesis, parametric design logic, and measurable mechanical performance.

In this context, the present study addresses this gap by proposing a biomimetic generative design framework that translates the interlocking mechanism of walnut shell sclereid cells into parametric design variables that systematically optimize interface geometry based on contact surface area. More specifically, a generative process is developed that emulates the identified biological design principles and produces interlocking structural blocks through algorithmic design tools. The mechanical behavior of 3D-printed specimens is evaluated through in-plane and out-of-plane flexural testing, examining the effect of key geometric parameters, such as protrusion height and distribution, on the interfacial contact area and structural performance. Finite element analysis (FEA) is also conducted to validate the experimental results.

## 2. Materials and Methods

### 2.1. Biomimetic Design Strategy

**Figure 1:**
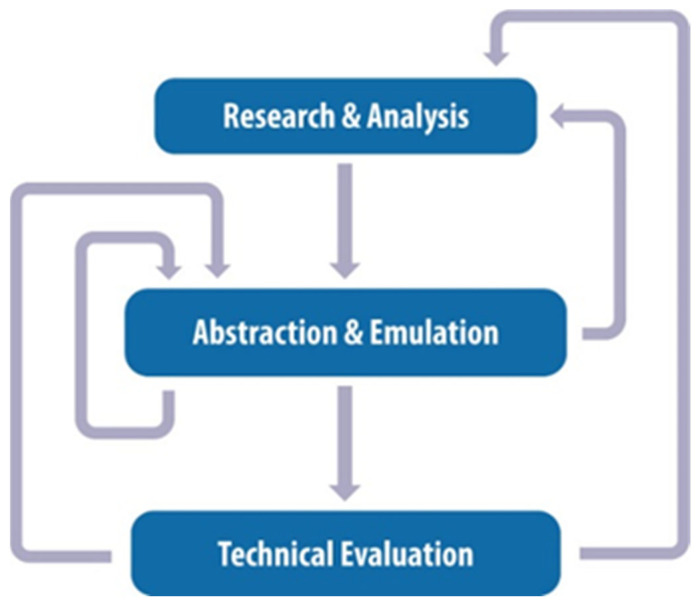
Conceptual biomimetic design framework illustrating the three interconnected stages of the proposed methodology: Research and Analysis, Abstraction and Emulation, and Technical Evaluation. The arrows indicate iterative feedback loops and knowledge exchange between stages. The diagram represents a non-linear methodological workflow, where insights from each stage may inform or refine the other three stages of the biomimetic design strategy and the feedback loops that connect and inform them in a non-linear and iterative process.

The present study follows a biomimetic design framework consisting of three interconnected stages, as illustrated in Figure 1. The first stage, Research and Analysis, focuses on biological investigation aimed at identifying relevant biological models and functional strategies. The second stage, Abstraction and Emulation, involves the systematic translation of these biological principles into parametric and generative design models through computational modeling and evolutionary optimization. The final stage, Technical Evaluation, comprises the fabrication of physical prototypes using additive manufacturing and the assessment of their mechanical performance through experimental testing. These stages form a non-linear and iterative methodological framework, in which knowledge and design insights obtained at any stage may inform, refine, or recontextualize the others. The bidirectional connections shown in Figure 1 therefore represent conceptual feedback and iterative refinement processes [27,55,56].

### 2.2. Research and Analysis

In the first stage of the biomimetic design process, research in the field of biology was conducted in order to identify potential design solutions in nutshells. Specialized databases, like AskNature, were used in the process. The walnut shell was shown to consist of a highly intricate shell structure that could provide meaningful design concepts in the fields of architecture and engineering. The walnut is the one-seed fruit of the tree *Junglas regia*, which is the most common nut tree in the world. Originally native in the region extending from the Balkans to Caucasus and southwest/central Asia, it is, nowadays, extensively cultivated across the globe between 10° and 50° northern latitude [57,58]. Specimens of mature walnut seeds were collected at Kroussia mountain, Kilkis region, Greece.

The Struers Minitom (Struers S.A.S., Champigny sur Marne, France) cutting machine was used to cut the walnut shell and prepare specimens for microscopy analysis. Initial analysis was conducted at the digital microscope Dino-Lite Pro HR—AD7013MZT (Dino-Lite Europe, Almere, The Netherlands). Subsequently, shell specimens were coated with gold (Au) particles using a Quorum SC7620 Sputter Coater (Quorum Technologies, Laughton, UK) to minimize the damage caused by electrical charging and improve imaging. They were fixed on double-sided carbon tape and further analysis of the shell’s microstructure was performed using a Phenom ProX Desktop Scanning Electron Microscope (SEM) (Thermo Fisher Scientific, Waltham, MA, USA).

### 2.3. Abstraction and Emulation

Computer-aided design (CAD) software Rhinoceros 3D (v.Rhino 7, 7.1.20343.09491, Robert McNeel & Associates, Seattle, WA, USA), in conjunction with the visual programming environment Grasshopper 3D (v.1.0.0007, Robert McNeel & Associates, Seattle, WA, USA), was used for the design transfer of the biological design concept identified in the walnut shell. In addition, the plug-in Galapagos, an evolutionary solver that enables the application of evolutionary algorithms by non-programmers, was implemented for the optimization of the biomimetic design [59]. It is tool that has been proven to efficiently apply evolutionary algorithms to optimize geometric, architectural and engineering solutions in a parametric environment [60,61,62]. More specifically, Galapagos automatically maximizes or minimizes a specific design parameter, called “fitness”, by finding the best possible collection of design variables. The individual variables that are examined by the solver, are referred to as “genes” and the collection of variables is referred to as “genome”. The evolutionary solver strategically examines the fitness landscape (or model space) that is created by the genome. As the solver starts, it populates the landscape with a random collection of variable sets (genomes). It then evaluates the fitness of each genome, keeping the fittest and discarding the unfit ones. Since Generation 0 of the genomes was random, the solver proceeds to create Generation 1 through inbreeding of the best genomes according to Generation 0. The new population is no longer random and, as this process is repeated multiple times, an optimal solution to the fitness requirement is reached.

### 2.4. Technical Evaluation

Physical specimens were fabricated on a Creality Ender 3 Pro (Shenzhen Creality 3D Technology Co., Shenzhen, China) FFF technology printer using commercially available polylactic acid (PLA) filament (NEEMA3D, Petroupoli, Greece) with a nominal diameter of 1.75 mm and dimensional tolerance of ±0.05 mm. Based on the manufacturer’s technical datasheet, the material has a specific gravity of 1.24 g/cm^3^, melt flow rate of 9.56 g/10 min (210 °C/2.16 kg), tensile strength at yield of 70 MPa, tensile modulus of 3120 MPa, and a melting temperature of 210 °C ± 10 °C. The recommended printing temperature range is 180–210 °C. The slicing process was performed on the Ultimaker Cura slicer (v.4.9.1, Ultimaker, Utrecht, The Netherlands). This technology was chosen as the structures are characterized by a relatively simple geometry which could be fabricated quickly, economically, and accurately. Physical specimens were printed with a layer height of 0.28 mm, which represents a compromise between surface resolution and printing time, ensuring consistent layer adhesion. The outer wall thickness was set at 0.8 mm, which implies 2 wall lines, given that the nozzle size is 0.4 mm, ensuring sufficient stiffness of the protruding features. In addition, 50% infill density of the structure was selected with a zig-zag infill pattern to provide internal structural support while minimizing material usage and printing time. The printing speed was set to 25 mm/s for the outer and inner wall speed and 50 mm/s for the infill speed to enhance the dimensional accuracy of the protrusions. A printing temperature of 205 °C and a build plate temperature of 55 °C were selected, according to the manufacturer’s instructions for PLA filament. Additional supports were used to warrant successful printing of the specimens. In particular, a support pattern of lines with a support density of 10% was applied to facilitate their removal. A cooling fan (90%) and brim adhesion were used to improve dimensional stability and print reliability. The complete set of printer parameters can be found in Table 1.

**Figure 2:**
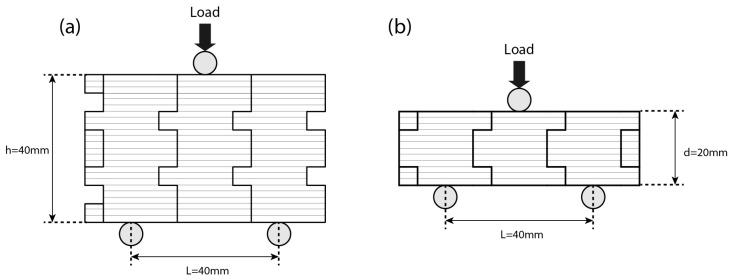
Mechanical bending tests of an array of interlocked structural elements with: (**a**) in-plane and (**b**) out-of-plane loads. The layers of the print are also distinguished which are in both cases transverse to the direction of the applied loads.

In order to investigate the mechanical behavior of the biomimetic interlocking structural elements, 3-point bending tests were performed on the Testometric M500-50AT system (Testometric company, Rochdale, UK) equipped with a 50 kN load cell at a constant strain rate of 5 mm/min, at in-plane and out-of-plane directions, on an array of three interlocked blocks, without an adhesive and according to the relevant literature [63,64]. Specimens fabricated with FFF are subject to anisotropy and print-orientation defects which are inherent to the manufacturing process and are known to influence their mechanical properties [65,66,67]. In the present study, these effects were controlled by maintaining identical printing parameters, material, printer, and print orientation for all specimens. All blocks were printed in the same orientation relative to the build plate, ensuring that the loading direction during three-point bending tests was consistent with respect to the layer deposition direction, as shown in Figure 2. As a result, anisotropy and potential manufacturing defects are assumed to affect all specimens in a comparable manner. In addition, the above tests were performed three times, to verify the results and derive a statistical model of the mechanical properties of the structure. The finite element (FE) software ANSYS 2025 R1 (ANSYS, Inc., Canonsburg, PA, USA) was utilized to delve into the mechanical characteristics of biomimetic interlocking structural elements. Employing an explicit dynamic analysis was crucial for accurately mimicking the structure’s significant distortions and bi-linear material behavior. The material properties assigned to the FE models correspond to those of the 3D-printed PLA used in the experiments. The elastic modulus and yielding point were taken from experimentally measured values and a linear elastic material model was adopted, as the primary objective of the simulations was to capture stiffness, stress distribution, and load transfer mechanisms prior to failure. To ensure that the results were not influenced by mesh density, a convergence assessment was conducted based on the strength of each interlocking structural element. This analysis revealed that stress convergence was reached with approximately 220,000 elements for each validation model, affirming the robustness of the findings. The validation and the effectiveness of the FEA were also confirmed by attaining the corresponding results in bending tests. All computational experiments were performed on a standard desktop workstation running Windows 10, equipped with an Intel Core i7 processor, 32 GB RAM and an NVIDIA GTX-series GPU.

## 3. Results

### 3.1. Morphological Analysis of the Walnut Shell

**Figure 3:**
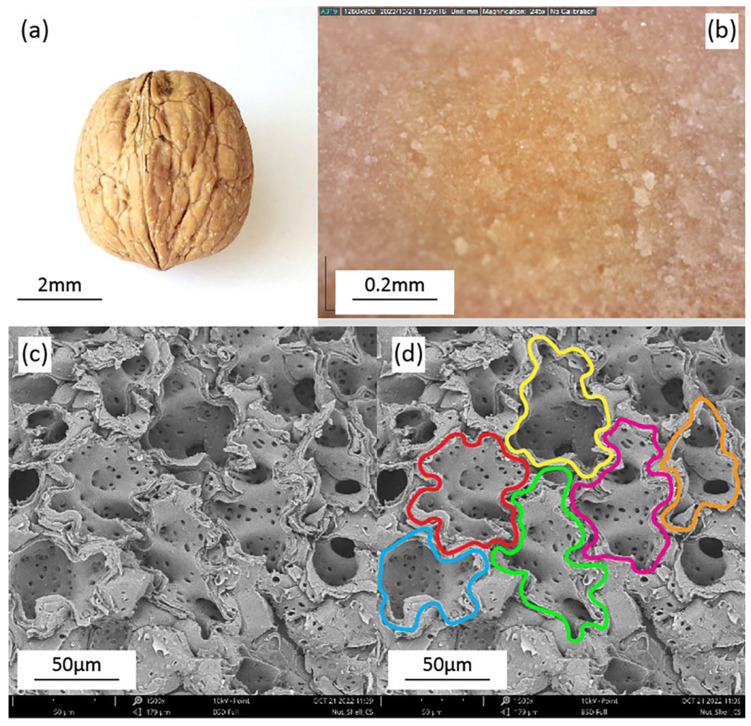
(**a**) The fruit of the walnut tree Juglans Regia; (**b**) the walnut shell as seen in optical microscopy; (**c**) the walnut shell under SEM, where the sclereid cells become discernible—the pores on the cell wall are also visible; (**d**) the individual sclereids are highlighted in order to emphasize the interlocking structure of the nutshell, where each cell develops protrusions in order to optimize its surface-to-volume ratio.

The walnut shell is characterized by a recently identified type of cell called sclereids, which develop protrusions as they mature, in an effort to optimize their surface area per unit volume [68,69]. As a result, they create a kind of three-dimensional puzzle structure, where they tightly interlock with their neighboring cells [59]. Mechanical interlocking can be considered as the main mechanism responsible for the high strength of the walnut shell [68]. The entirety of the cell wall is constructed from a single type of cell, the 3D puzzle sclereids, in contrast to shells of other species, like Macadamia or hazelnut, which use a combination of different cells, like fibers, isodiametric sclereids or sclerenchyma cells [37,69,70]. Microscopic images of the walnut shell and sclereid cells are illustrated in Figure 3b,c and the puzzle structure is highlighted in Figure 3d. The average size of each cell is 94.1 × 10 μm^3^ and the cell surface is 17.6 × 10 μm^2^. A mature cell also consists of around 12 lobes, with a solidity of 0.61 ± 0.06, which represents the ratio between cell volume and convex hull volume. Compared to a tetrakaidecahedron, which is the closest geometric shape, the cells are characterized by 30–40% more surface area per unit volume [33,68,69,70]. Apart from the protrusions, pores can also be identified on the cell wall, as seen in Figure 3c, a structural feature that enables the exchange of chemical substances during the development of the walnut fruit [63]. Studies on the chemical composition of the cell wall reveal that it mainly consists of lignin (over 50%), which is followed by cellulose at 25% and hemicelluloses at 22%, arranged in laminated layers [71,72]. Mechanical testing of walnut shells conducted by Antreich et al. shows significantly improved strength when compared to other nutshells, which can be attributed to the higher surface area and interlocking mechanism, where protrusions need to fracture in order to allow for the collapse of the overall shell [68]. The large surface of the sclereids constrains the cells together, significantly enhancing stress propagation, absorption capacity and resistance to localized failure [73,74,75].

### 3.2. Generative Design Process

The biological design strategy that was identified in the walnut shell was transferred to a novel design of interlocking structural blocks. To establish a direct link between the biological system and the design abstraction process, the key morphological features of the walnut shell were translated into corresponding geometric and parametric design principles. More specifically, the protrusions developed by sclereid cells to increase surface area per unit volume were interpreted as interfacial geometric features that enhance mechanical interlocking. The three-dimensional puzzle-like arrangement of the cells was translated into complementary protrusion patterns between adjacent blocks, enabling load transfer through distributed contact rather than localized interfaces. As a result, the biological principle of surface-driven interlocking was implemented in the generative model through the maximization of contact surface area as the primary fitness criterion. This mapping establishes a direct relationship between biological morphology and engineering performance.

**Figure 4:**
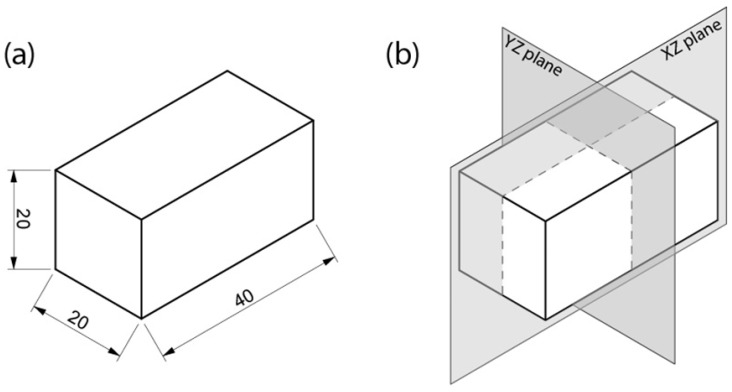
(**a**) The dimensions of the original block; (**b**) the symmetry planes of the protrusions in order to achieve optimal placement and load distribution.

More specifically, a generative algorithm was created that takes a basic structural element with predefined dimensions and volume and automatically generates protrusions on the contact surfaces between adjacent blocks in order to maximize the interface area and mechanically interlock them, while maintaining the original volume value. The objective function of the evolutionary optimization was defined as the maximization of the total contact surface area between complementary block interfaces, as increased contact area is directly associated with enhanced load transfer and mechanical interlocking. A standard block with flat surfaces was taken as the basis of the optimization process. Since the design algorithm is parametric, any dimensions can be given to the block. However, for the purposes of this study, a block of 40 × 20 × 20 mm, as illustrated in Figure 4a, was selected to serve as the baseline, following the typical ratio of 2:1:1 usually found in the dimensions of standard blocks [76].

**Figure 5:**
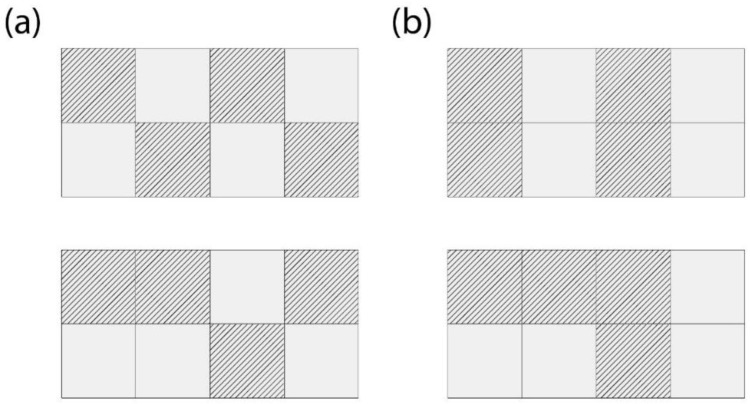
Examples of protrusion patterns: (**a**) Checkerboard patterns lead to open BREPs and should be avoided; (**b**) Non-checkerboard patterns that create closed BREPs are better suited for an interlocking interface.

**Figure 6:**
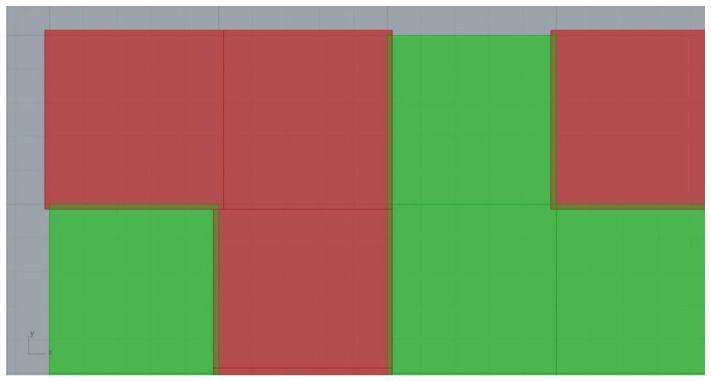
The XY dimensions of the gaps (red) between the protrusions (green) are offset by a factor of 1.06 and translated to 0.3 mm on each side. The overlapping volume is removed from the protrusions to allow for seamless interlocking of the printed specimens.

In addition, it was deemed necessary to apply further design constraints to the requirements of the generative algorithm for computational, manufacturability and mechanical performance purposes. Initially, a symmetrical pattern of protrusions was preferred, with two levels of symmetry along the XZ and YZ planes, as shown in Figure 4b, for more efficient element placement, uniform load distribution and faster computation times [76]. Checkerboard patterns, such as those illustrated in Figure 5a, were also avoided, as they would create open Boundary Representations (BREPs) in the geometric plane, which could lead to problematic interlocking [77]. Arrangements that do not form a checkerboard, similar to those depicted in Figure 5b, would prevent this problem and lead to optimal interlocking. A small offset in the dimensions of the protrusions is also required to allow for seamless interlocking of the elements, the extent of which depends on the method and material of construction [78,79,80]. As the FFF 3D printing technology with PLA material was chosen for this study, displacement with a factor of 1.06 was defined in the XY dimensions of the gaps between the overlapping projections, where the overlapping volume is then subtracted from the main volume of the protrusions, as shown in Figure 6. This translates to 0.3 mm on each side of the protrusions which falls within the ideal offset range that is recommended for PLA interlocking joints [75,80,81].

**Figure 7:**
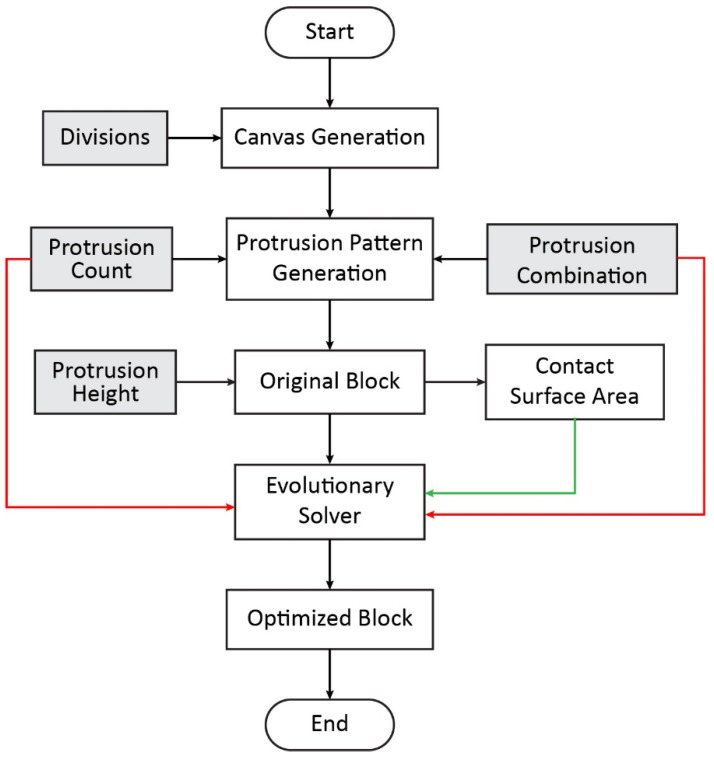
Top-level parametric data-flow diagram of the generative design process in Grasshopper. The diagram shows the relationships between user-defined parameters, genome variables, geometric generation, and fitness evaluation. User-defined parameters are shown in light gray, while genome and fitness parameters are highlighted in red and green, respectively.

A generative algorithm was designed with the aid of the Grasshopper visual programming environment, which autonomously optimizes the contact surface of interlocking blocks, while maintaining a constant volume. Figure 7 provides a top-level parametric data-flow representation of this process. The figure summarizes the main parameter groups, geometric generation stages, and fitness evaluation components, as well as their data dependencies within the parametric model. It serves as a conceptual overview of how user-defined parameters, genome variables, and the evolutionary solver interact within the Grasshopper environment.

The variables “protrusion count” and “protrusion combination” constitute the genome of the evolutionary solver and control the spatial configuration of protrusions on the block interface. More specifically, “protrusion count” determines the number of active protrusions within the predefined canvas, while “protrusion combination” defines their specific arrangement. These parameters directly influence the resulting geometry of the interlocking interface. The fitness function of the optimization is defined as the total “contact surface area” between complementary block interfaces, which is maximized during the evolutionary process. The allowable range of the variables is defined by an initial 2 × 4 grid mirrored along the XZ and YZ planes, resulting in 32 possible positions. This configuration was selected to balance exploration of the design space with computational efficiency, manufacturability and structural consistency.

**Figure 8:**
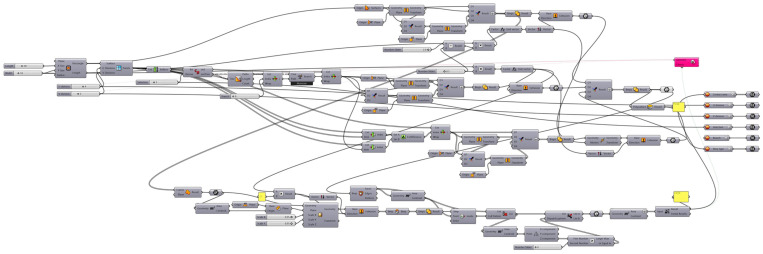
Screenshot of the complete Grasshopper definition used to implement the generative algorithm. The figure documents the actual parametric model as executed within the visual programming environment and serves as an implementation reference.

**Figure A1:**
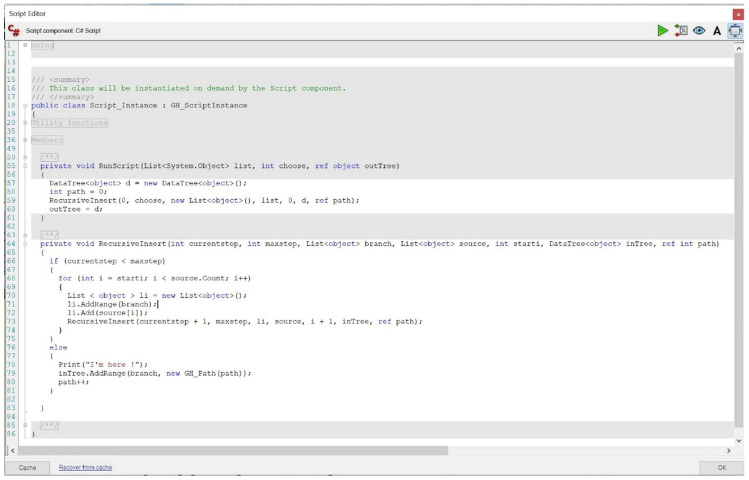
The custom python script that enables the selection of a set of protrusions and all their possible combinations in the form of a data tree.

At the top algorithmic level, a planar surface is initially created and divided into a grid of potential protrusion locations. A pattern is then generated using a custom Python script (IronPython v.2.7.9.0), which enables the selection and combination of protrusions on the defined canvas (Figure A1 of Appendix A). Based on this pattern, an interlocking geometry is constructed, while a user-defined height parameter is assigned to the protrusions. The total contact surface area of the resulting geometry is subsequently calculated and used as the fitness function of the optimization process. The evolutionary solver Galapagos iteratively evaluates different configurations of protrusion arrangements and identifies those that maximize the contact surface area. The final optimized geometry is derived through this automated, iterative process. The complete implementation of the algorithm within the Grasshopper environment is presented in Figure 8. Increasing the height of the protrusions leads to an increase in contact surface area. For this reason, this parameter was excluded from the optimization process and examined separately in the analysis.

**Figure 9:**
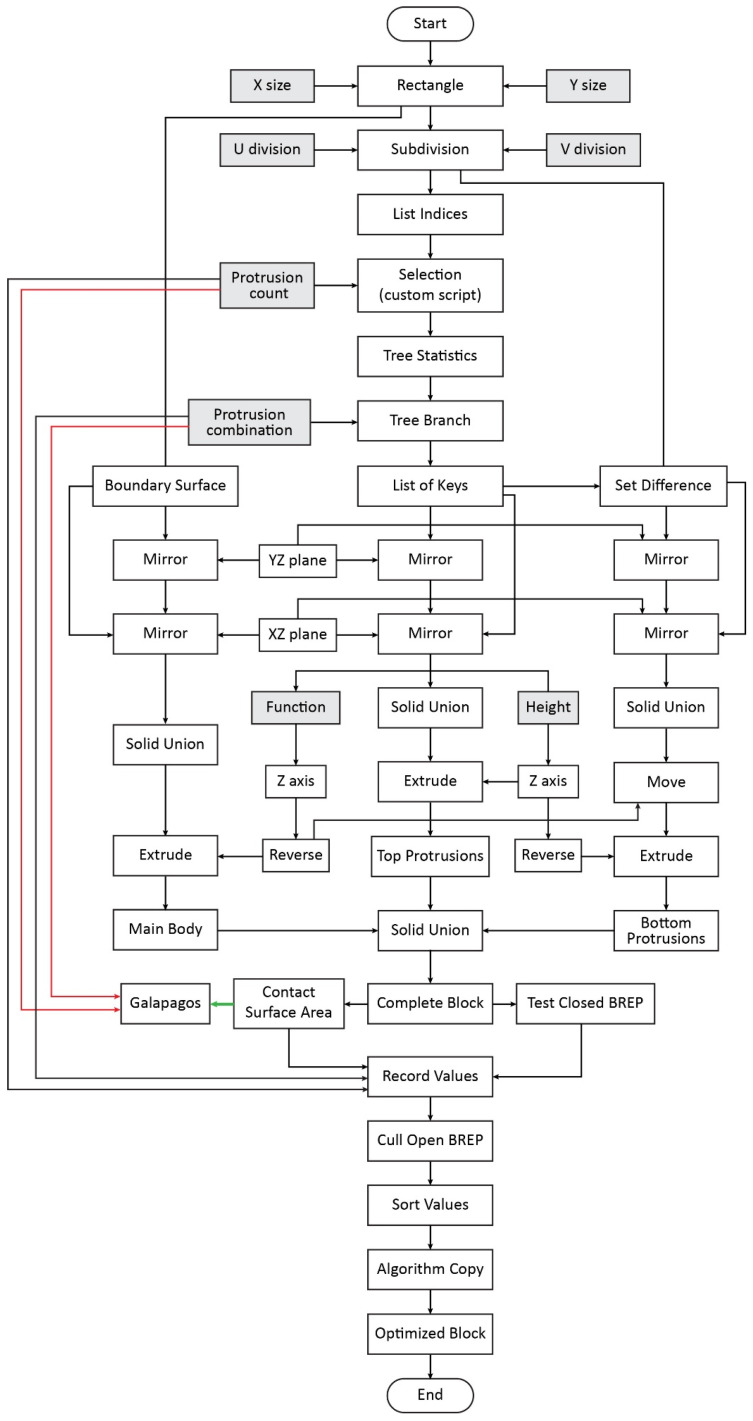
Second-level parametric workflow diagram providing an analytical abstraction of the Grasshopper definition shown in Figure 8. The diagram decomposes the generative process into its main geometric, parametric, and data-processing components, illustrating how inputs, transformations, and fitness evaluation interact within the model. User-defined parameters are seen in light gray boxes; the genome and fitness parameters are highlighted with red and green lines, respectively.

A more detailed description of the generative algorithm is provided by the second-level parametric workflow diagram shown in Figure 9. This figure offers an analytical abstraction of the Grasshopper definition, decomposing the model into its principal geometric operations, parametric inputs, and data-processing steps. It is intended to clarify how data and geometric entities propagate through the parametric system and how solutions are evaluated by the evolutionary solver, rather than to represent a procedural or decision-based execution sequence.

At this level, a rectangle is initially drawn with custom dimensions, which is then subdivided into panels, which will form the basis of the protrusions. An indices list of the panels is extracted, and the custom python script is implemented which selects a specific number of indices and all the possible combinations that can be derived from the specific set of indices. The output data tree is analyzed, and a specific branch is selected which corresponds to an individual combination of panel arrangement. In order to achieve the symmetry requirement, the set of panels is mirrored along the YZ plane, and the two sets are mirrored once more along the XZ plane. The now symmetric panels are merged and extruded along the Z axis, at a user-defined height. The top protrusions of the interlocking blocks have been successfully created at this point. Boundary surfaces are created out of the subdivision panels, which are mirrored along the YZ and XZ planes and merged. The resulting rectangle is extruded to form the main body of the block at a specific height, which is expressed as a function of the top protrusion’s height in order to maintain a constant volume for the block. The bottom protrusions are generated next. The set difference between the item indices of all the panels and the item indices of the top panels is extracted. The output panels, which are essentially complementary to the top panels, will form the basis of the bottom protrusions of the interlocking block. Once again, the panels are mirrored along the YZ plane first, and along the XZ plane secondly. The symmetric panels are merged and moved along the Z axis at a value equal to the height of the main body. Once in place, they are extruded at a height equal to the height of the top protrusions, and the bottom ones are formed. At this point, the complementary top and bottom protrusions are merged along with the main body into a single solid BREP, which is then tested to check whether it is open or closed. Open BREPs indicate a checker-style arrangement. The contact surface of the block is also calculated as the sum of the area of the top and bottom surfaces of the block after the generation of the keys.

The evolutionary solver “Galapagos” is implemented in order to optimize the contact surface area of the block, which is defined as the fitness function of the solver. The “Protrusion selection” and “protrusion combination” parameters are set as the genomes of the solver, as they fully describe the spatial configuration of the interlocking features. The contact surface area of the blocks is set as the fitness input, which the solver attempts to maximize. The number of individuals per generation of solutions was set to 100, a value selected to balance exploration of the solution space and computational cost. The multiplication factor for Generation 0 was set at five (5 × 100 = 500 solutions in the first generation), in order to increase initial genetic diversity and avoid premature convergence. Then, 5% of the better solutions were carried over to the next generation and 75% of inbreeding between them was conducted in each generation. These values provide a healthy balance of inbreeding between already identified optimal and new random solutions, so that a large space of parameter combinations could be explored. The maximum stagnant generations before the solver aborts were set at 50, which defines the convergence criterion of the solver and ensures termination once no further improvement in the fitness value is detected. The values of the “contact area”, “Protrusion selection”, “protrusion combination” and “BREP type” are recorded as the solver is running and internalized into a numeric set of parameters. The values of the respective parameters were culled based on the closed or open state of the BREP, which are recorded as 0 and 1 (false and true, respectively), ensuring geometric validity. The remaining value sets are sorted based on the area of the contact surface of the respective design iteration. As such, the optimal design solutions can be identified. The parameters of “Protrusion selection” and “protrusion combination” are fed into a copy of the algorithm to generate the optimized design solution. Table 2 shows the defined parameters of the evolutionary solver.

**Figure 10:**
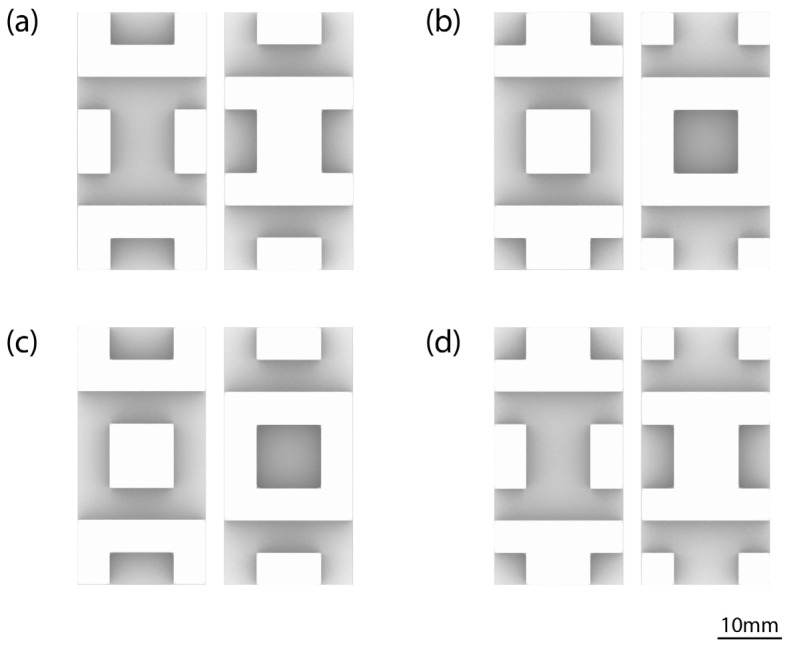
The four optimal protrusion arrangements for an 8 × 4 canvas of the top surface of the block along with the corresponding pattern of the bottom surface, as generated by the evolutionary algorithm (all models are shown on the same scale): (**a**) first variant, (**b**) second variant, (**c**) third variant, and (**d**) fourth variant.

The end result is a generative algorithm that can autonomously generate the optimal protrusion arrangement and produce an optimal interface for mechanically interlocked blocks, while preserving the original material volume, adhering to the biological principle that was identified in the walnut shell. The arrangement is also symmetrical and non-checkerboard styled for optimal block placement and load distribution. As the algorithm is parametrized, it can be applied to blocks with variable dimensions and with variable canvases of potential protrusions. The described evolutionary algorithm generated 5301 total iterations before it automatically stopped, with 4131 of them producing closed BREPs. When these are sorted on the basis of the contact areas of the blocks, four optimal design solutions are extracted. A second run of the evolutionary solver was performed with identical parameters for verification purposes. A total of 5601 design iterations were examined and 4590 closed BREPs were produced. The same four optimal protrusion arrangements were generated. In both cases, when the design solutions are sorted based on the “contact area” parameter of the blocks, four optimal design solutions are extracted, with a total area of 2800 mm^2^, indicating convergence and repeatability of the optimization process. The optimal patterns, as determined by the evolutionary solver, consist of 16 total protrusions for both the upper surface and the complementary lower surface, arranged in the patterns illustrated in Figure 10. These are derived from the selection of four initial protrusions (parameter “parameter count”) which are mirrored along the XZ and YZ planes.

**Figure 11:**
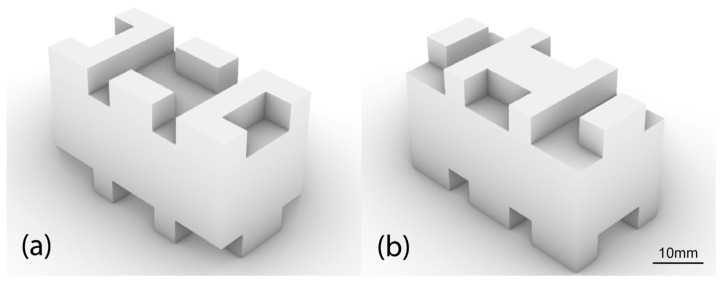
The design variation in the optimized biomimetic interlocking structural element selected for further study. The complementary protrusions are distinguished: (**a**) on the upper surface of the block and (**b**) on the lower surface of the block.

Of the four optimal design solutions, the design variant distinguished in Figure 10a was selected for further analysis and technical evaluation of the biomimetic building block. The set of geometric parameters of the optimized design solution is listed in Table 3, as generated by the evolutionary solver based on the fitness function and genome criteria. It should be noted that the reported length of 20 mm and width of 10 mm correspond to final block dimensions of 40 mm and 20 mm, respectively, as the original plane is mirrored twice. The same applies to the protrusion count, where four initial protrusions correspond to 16 protrusions in the final pattern. The protrusion height was manually set at 5 mm for the protrusions (“protrusion height”) and 15 mm for the main body (“main body height”). These values were selected to provide sufficient interlocking depth and mechanical engagement while maintaining a constant block volume. As mentioned earlier, the effect of protrusion height on the structure will be studied separately. A 3D model of the particular design solution is shown in Figure 11, illustrating the protrusion pattern on the upper and lower surfaces of the block.

### 3.3. Mechanical Characterization of the Interlocking Blocks Assisted by FEA

The structural element that served as the basis of the optimization process (Model 0) is characterized by 0 overhangs and a total interface area of 1600 mm^2^. The optimized model (Model 3) has a total contact area of 2800 mm^2^, which is 75% larger than the original model. Two more models (Models 1 and 2) are obtained by progressively removing overhangs from Model 3 and subsequently reducing the total contact area. Model 1 has four protrusions and an interface area of 2000 mm^2^ (20% larger than Model 0), while Model 2 has eight protrusions and an interface area of 2400 mm^2^ (50% larger than Model 0). All blocks have the same volume of 16 cc.

**Figure 12:**
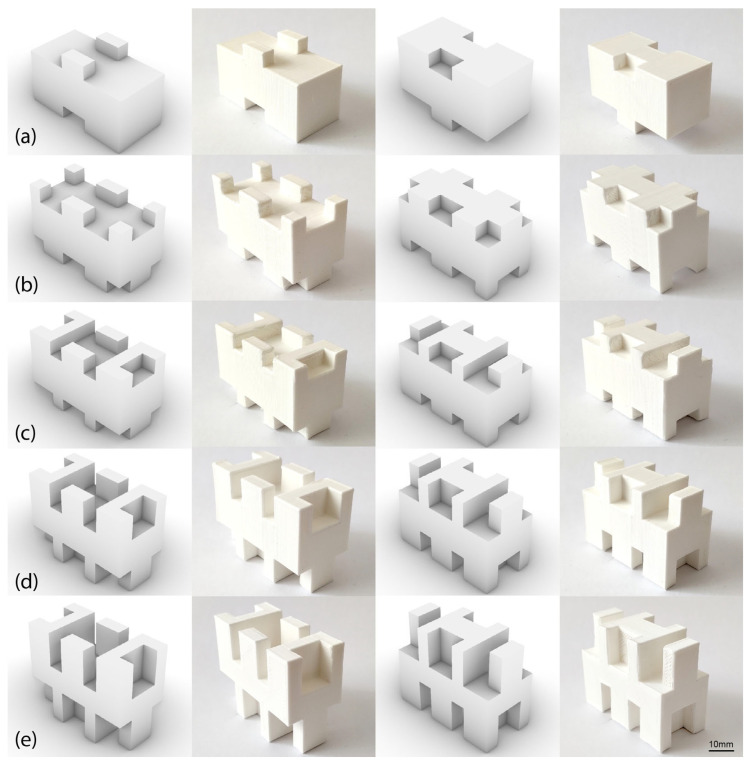
Top and bottom layouts for the digital and printed models of biomimetic interlocking block of: (**a**) Model 1, (**b**) Model 2, (**c**) Model 3 (optimized), (**d**) Model 4 and (**e**) Model 5. All models are shown at the same scale for proper comparison.

As mentioned previously, another way to increase the interlocking interface area is by increasing the height of the protrusions. Two more design variants (Models 4 and 5) were created based on the optimized Model 3. In Model 4, the height of the overhangs increased to 8.5 mm, with a consequent increase in the interface area to 3640 mm^2^, an increase of 127.5% relative to the area of Model 0. In Model 5, the height of the protrusions was increased to 12 mm and the contact area to 4480 mm^2^, equivalent to a 180% increase. The parametric block was designed to maintain its original volume despite the changes in protrusion height. The geometric characteristics of all the models can be found in Table 4 and the corresponding digital models, along with the respective 3D-printed specimens, in Figure 12.

**Figure 13:**
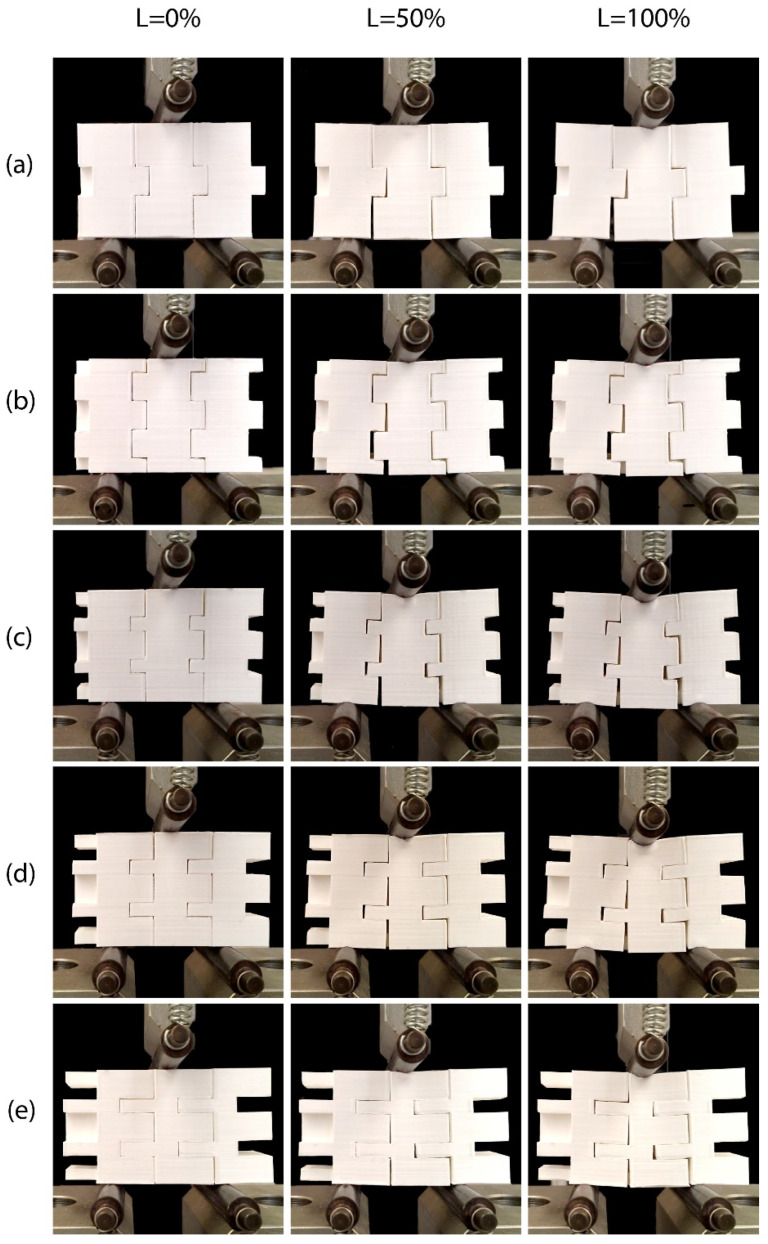
Behavior at 0%, 50% and 100% of the Lmax displacement during the in-plane flexural test of an array of three specimens of: (**a**) Model 1, (**b**) Model 2, (**c**) Model 3, (**d**) Model 4, and (**e**) Model 5.

**Figure 14:**
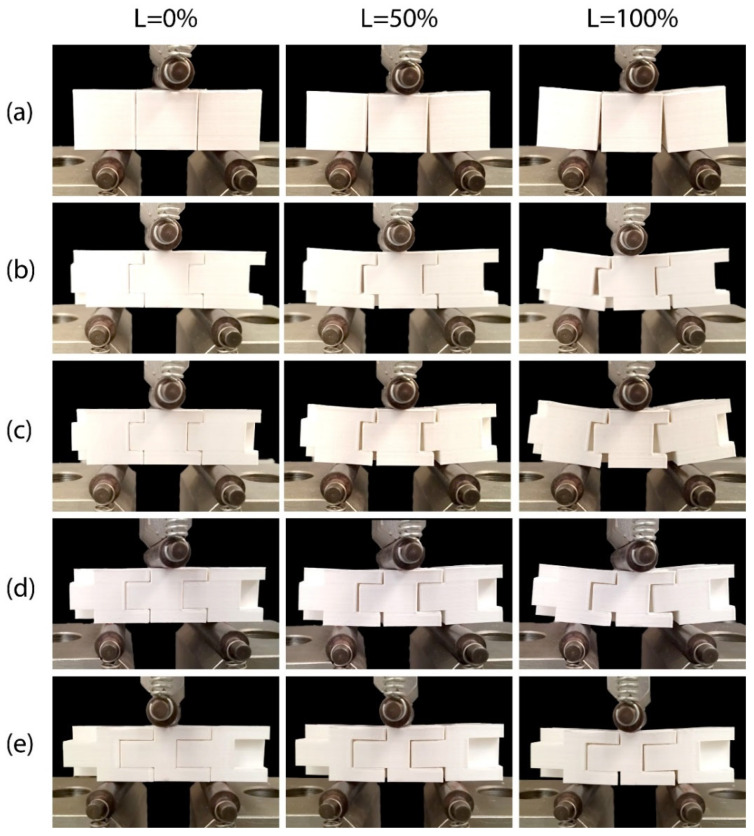
Behavior at 0%, 50% and 100% of Lmax displacement during out-of-plane flexural test of an array of three specimens of: (**a**) Model 1, (**b**) Model 2, (**c**) Model 3, (**d**) Model 4, and (**e**) Model 5.

**Figure 15:**
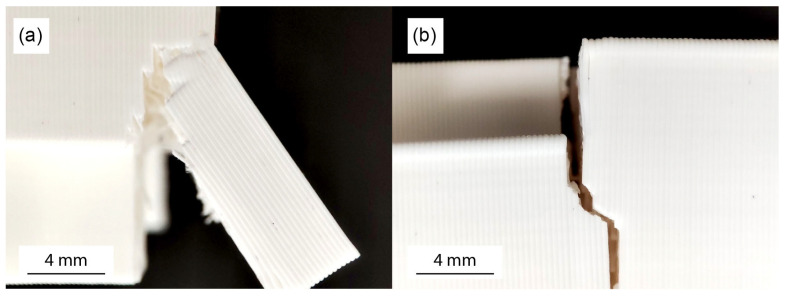
Typical failure of interlocking blocks occurs due to concentrated loads at the base of protrusions, leading to crack propagation along the print layers: (**a**) fractured specimen showing material separation, (**b**) crack propagation path along the printed layers.

Figure 13 and Figure 14 illustrate frames of the in-plane and out-of-plane three-point bending tests, respectively, of a three-block array of the printed models. The first column in the images depicts the specimens at the start of the tests, the second at 50% of the maximum L_max_ displacement and the third at 100% of the maximum displacement. In both the in-plane and out-of-plane tests, similar mechanical behavior is observed between the different specimens. The absence of adhesive material allows slight separation of the blocks at the bottom of the array [47]. Increasing the number of protrusions, as well as increasing their height, seems to prevent this behavior through more effective interlocking of the structural elements. The failure of the blocks can be traced to concentrated dynamic loads developed at the base of the protrusions, which lead to their fracture [63]. These cracks then propagate along the printed layers, resulting in their delamination [67,82]. Figure 15 illustrates typical failures observed in the blocks. At this point, the importance of the direction of specimen printing is verified, as choosing a direction parallel to the developing loads would result in faster collapse of the protrusions and a reduction in the overall strength of the blocks [67].

**Figure 16:**
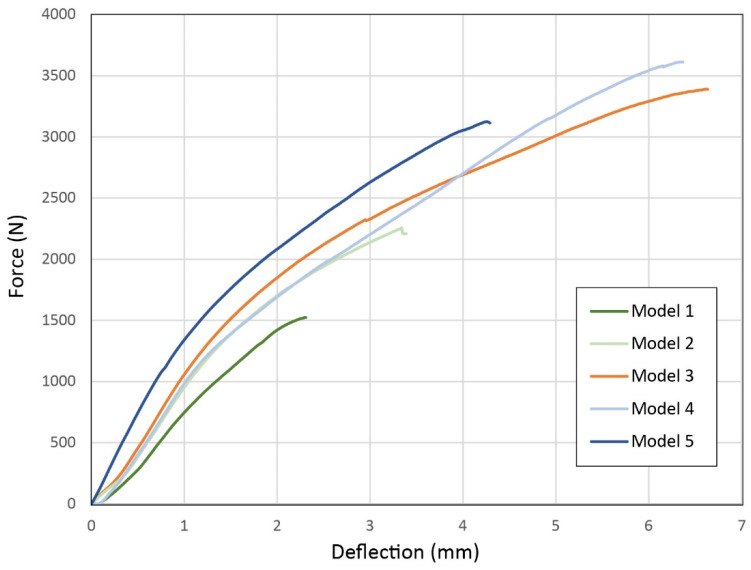
Force-deflection curves of in-plane bending tests of biomimetic interlocking structural elements.

The in-plane force-deflection curves of the biomimetic interlocking building blocks are shown in Figure 16. The maximum in-plane load that the three-specimen array of the generatively optimized Model 3 can withstand is 3526.75 ± 193.96 N. There is a substantial decrease in the maximum load in Model 2, where it is equal to 2300.45 ± 64.56 N. The same decreasing trend is also observed in Model 1, where the maximum load recorded is 1651.86 ± 180.52 N. It becomes evident that as the interface area of the interlocking biomimetic blocks decreases with the removal of protrusions, their in-plane bending load resistance decreases simultaneously. The optimized placement of the protrusions appears to enhance the mechanical properties of the blocks. Model 4 is characterized by a maximum bending load of 3586.25 ± 38.11 N, which is marginally increased compared to Model 3. However, Model 5 shows a decrease in the maximum load, which is equal to 3012.70 ± 157.26. It becomes evident that increasing the interface area by increasing the length of the overhangs can slightly benefit the strength of the structure up to a point (from 5 mm to 8.5 mm). However, an excessive increase in length (12 mm) has the opposite effect as it leads to a reduction in the maximum load that the structure can withstand. This effect is in agreement with the relevant literature and can be attributed to the development of stress concentrations at the base of the protrusions due to ineffective load paths which lead to rotational effects and root shearing of protrusions, exacerbated by layer delamination of the 3D-printed structure [49,83,84,85,86,87]. The total maximum in-plane loads are summarized in Table 5.

**Figure 17:**
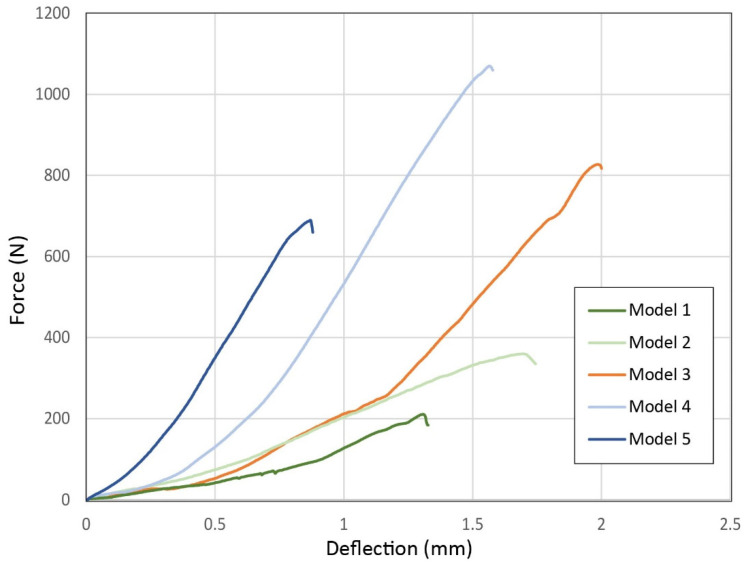
Force-deflection curves of out-of-plane bending tests of biomimetic interlocking structural elements.

Figure 17 illustrates the force-deflection curves obtained from the out-of-plane three-point bending tests of the interlocking blocks. First of all, it is observed that the maximum loads of all blocks are significantly reduced compared to the in-plane tests. The maximum load recorded for Model 3 is 865.50 ± 54.59 N. The maximum loads for Models 1 and 2 are 203.35 ± 10.39 N and 369.55 ± 10.11 N, respectively. There is a reduction in the strength of the blocks as the interface area is reduced through the removal of protrusions, similar to that recorded in the in-plane tests. Therefore, it is concluded that the optimized protrusion patterns also significantly enhance the out-of-plane mechanical properties of the blocks. The maximum loads for Models 4 and 5 are 996.10 ± 117.24 N and 823.15 ± 86.20 N. There is a slight increase in the strength of the structure as the height of the projections increases to 8.5 mm. However, as in the in-plane tests, this behavior is reversed from one height onwards (12 mm), resulting in a significant decrease in the strength of the structure. The values of the out-of-plane maximum loads are recorded in detail in Table 5. From the large variation observed in the slope of the curves, it is also evident that the increase in the length of the protrusions results in a significant increase in the stiffness of the specimens when subjected to out-of-plane loads.

The observed standard deviation values are attributed to the inherent variability of the additive manufacturing process, particularly in FFF printing, where factors such as layer adhesion, minor geometric deviations, and interfacial contact conditions between blocks can influence the mechanical response. Additionally, the interlocking behavior introduces sensitivity to local contact interactions and frictional effects, which may vary slightly between specimens. Despite this variability, the overall trends remain consistent across all configurations, confirming the reliability of the results and the validity of the observed relationships between geometric parameters and mechanical performance.

It becomes evident from the above results that the generative algorithm, based on the biomimetic principle identified in the nutshell, produces interlocking building blocks with improved mechanical properties. The geometries identified as optimal by the evolutionary solver are characterized by protrusion patterns that establish continuous load transfer paths across the interface, rather than relying on discrete contact points. Under bending, this results in a more uniform distribution of stresses along the interlocking surfaces, reducing peak stress concentrations and delaying the onset of localized failure. The generated protrusion patterns enhance resistance to both in-plane and out-of-plane bending loads, as the applied forces are distributed across multiple contact regions and transferred through a network of interacting protrusions. In contrast, suboptimal geometries with fewer, poorly distributed, or excessively slender protrusions tend to localize stresses at isolated contact zones, leading to premature failure, particularly near the base of the protrusions where stress intensification and bending-induced moments are highest.

Furthermore, the interlocking configurations enable progressive mechanical engagement during loading, as additional protrusions come into contact under deformation. This results in a gradual increase in load-bearing capacity and contributes to improved structural stability. Frictional interactions between contacting surfaces also play a significant role, as they resist relative sliding between adjacent blocks and enhance the overall stiffness of the system. The influence of protrusion height further highlights the balance between increased interfacial contact and structural stability. While greater protrusion height increases the effective contact area and improves mechanical interlock, it also introduces higher bending stresses at the base of the protrusions, which can lead to reduced performance beyond a certain threshold. These observations indicate that the superior performance of the optimized geometries results from a combination of distributed load transfer, progressive interlocking engagement, and frictional resistance, all of which are controlled through the geometry of the interface.

**Figure 18:**
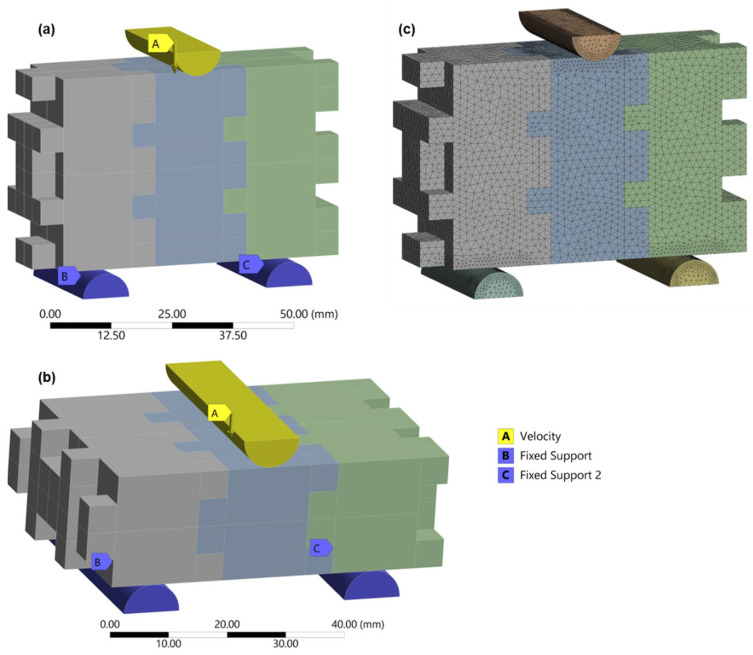
Boundary conditions applied to (**a**) in-plane and (**b**) out-of-plane biomimetic interlocking structural elements, while (**c**) demonstrates the typical meshing utilized in the FE analysis.

A computational model was employed to assess how 3D-printed biomimetic interlocking structures respond to compression-induced stress. The biomimetic structures underwent incremental vertical velocities applied to the top plate of the steel pin, while the corresponding reaction forces were recorded at the fixed-support bottom boundary. The real vertical displacement values were determined from experimental outcomes and imported into the FE model. Boundary conditions and loading configurations in the FE simulations were defined to replicate the experimental three-point bending tests as closely as possible. The span length, support conditions, and loading point location were identical to those used experimentally. Supports were modeled as frictionless constraints allowing rotation, while a vertical displacement-controlled load was applied at the mid-span. Contact interactions between interlocking surfaces were modeled using surface-to-surface contact with finite sliding, enabling realistic simulation of mechanical interlocking and load transfer between blocks. The boundary conditions for both in-plane and out-of-plane loading conditions are illustrated in Figure 18a,b. Tetrahedral elements were used for the blocks (in and out of plane), while the upper bending pin and the supports at the bottom were also discretized by tetrahedral elements, as shown in Figure 18c.

**Figure 19:**
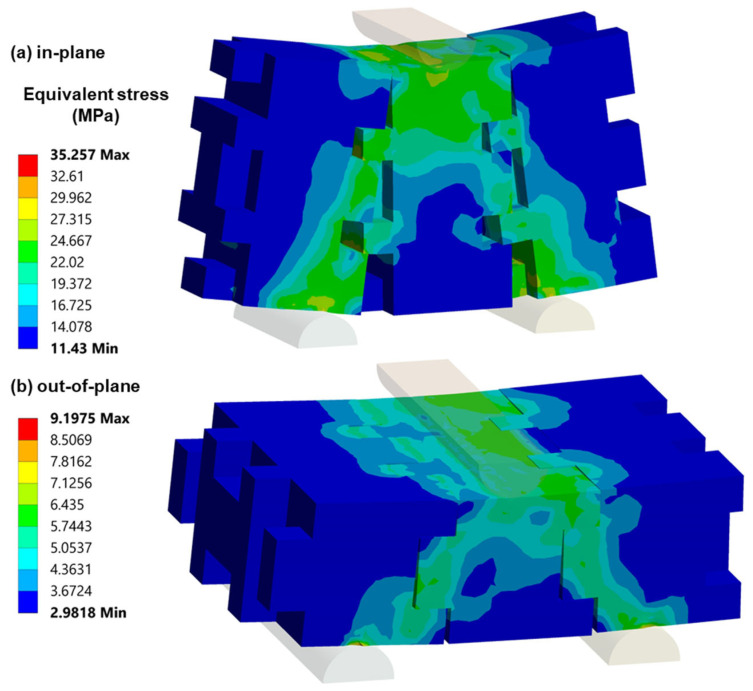

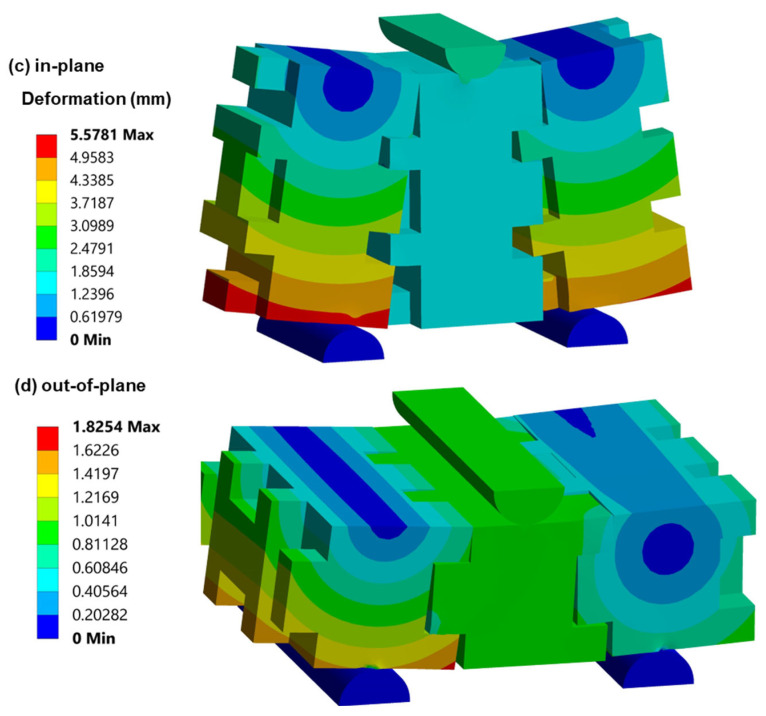
Equivalent stress distribution of the optimal biomimetic interlocking structural element (Model 3) under bending load, (**a**) in-plane and (**b**) out-of-plane. Directional deformation of the optimal biomimetic interlocking structural element (Model 3) under bending load, (**c**) in-plane and (**d**) out-of-plane.

The finite element (FE) results depicted in Figure 19 mirror the experimental findings of the study, confirming the enhanced mechanical performance of biomimetic interlocking building blocks with optimized protrusion patterns. The FE analysis reveals that the distribution of loads among the protrusions and the optimized block interface significantly improves both in-plane and out-of-plane bending loads, aligning with the experimental observations. Additionally, the FE simulations highlight the detrimental effect of excessive protrusion height on specimen strength, reinforcing the importance of balanced design for optimizing mechanical properties. In Figure 19a,b, corresponding to stress levels a and b, contour distributions illustrate stress patterns, while Figure 19c,d demonstrate deformations that closely match experimental results, with Figure 19a,c representing in-plane conditions, and 19b and 19d depicting out-of-plane scenarios. In order to establish a direct correlation between the finite element results and the experimental findings, the forces were derived from the FEA stress fields based on a geometrically defined load-bearing interlocking region. The FE model was driven by displacement boundary conditions corresponding to the experimentally measured maximum deflection, ensuring identical deformation states between simulation and experiment. For the representative case of Model 3, the maximum stresses obtained from the FE analysis (~35 MPa for in-plane and ~9 MPa for out-of-plane loading) correspond to forces of approximately 3500 N and 900 N, respectively. These values are in very close agreement with the experimentally measured forces (3526.75 ± 193.96 N for in-plane and 865.50 ± 54.59 N for out-of-plane loading), with deviations below 5%. Conclusions drawn from mechanical flexural test results indicate that combining computationally generated (FEA) bending test data with actual measurements could be an efficient approach for characterizing the mechanical deformation behavior of 3D-printed biomimetic configurations.

Beyond the observed improvements in mechanical performance, the proposed approach enables the systematic design and optimization of interlocking interfaces based on quantifiable geometric parameters. Unlike conventional interlocking systems that rely on predefined geometries, the presented framework enables the exploration of a broader design space and the identification of configurations that enhance load transfer and stress distribution through increased interfacial contact. Furthermore, the results establish a clear relationship between geometric parameters and mechanical behavior, providing actionable design guidelines for the development of interlocking systems. In particular, the number and distribution of protrusions directly influence load transfer efficiency, while their height affects both the extent of mechanical interlocking and the development of stress concentrations. The findings highlight the critical role of interface geometry in controlling structural behavior. This integration of biomimetic principles with generative design and experimental validation offers a transferable methodology for engineering applications where efficient load distribution and modular assembly are required.

At the same time, certain limitations of the proposed approach should be acknowledged. The experimental results are based on FFF-fabricated PLA specimens and therefore reflect the inherent material anisotropy and process-related variability of additive manufacturing. In addition, the study focuses on relatively small-scale specimens, and further investigation would be required to assess the scalability of the proposed design to larger structural applications. Factors such as manufacturing time, material performance, and contact conditions between elements may influence behavior at larger scales. Lastly, investigation into suitable construction materials and methods for large-scale applications is necessary to ensure structural reliability, durability, and feasibility in real-world building environments.

## 4. Conclusions

The walnut shell is made up of special cells, called sclereids, which form protrusions to optimize the area of their contact surface with neighboring cells, thus forming a three-dimensional puzzle where the cells mechanically interlock with each other, and giving the shell high strength. This biological design strategy served as an inspiration for the design of interlocking structural elements without the use of adhesive material. A generative algorithm was formulated using the Galapagos evolutionary solver, which generates protrusions on the structural element’s contact surfaces, optimizing its area while keeping the original block volume constant. Additional design constraints were applied, such as symmetric overhang patterns and avoidance of checkerboard-like patterns for more effective placement, load distribution and computation time. The solver examined a very large number of design variations, from which four optimal ones were obtained.

Of these four models, one was selected for further evaluation of the biomimetic structure. A series of models were generated by removing protrusions and consequently reducing the interface area, as well as by increasing the height of the protrusions and subsequently increasing the area. These five models were examined in three-point, in-plane and out-of-plane bending tests on arrays of three interlocked blocks. The physical specimens of the models were printed on an FFF printer with PLA material with different orientations so that the print layers remained transverse to both in-plane and out-of-plane bending loads. Some compromises were chosen in print quality due to the large number of specimens.

Similar mechanical behavior is observed in in-plane and out-of-plane tests. Minimal separation occurs at the bottom of the block array due to the non-use of adhesive; however, increasing the number and height of the projections produces more effective interlocking. Failures are mainly observed at the base of the overhangs, where there is a higher concentration of loads, which are propagated along the print layers. The maximum in-plane load that the structure can withstand decreases as the interface area is reduced by removing overhangs. Instead, it increases marginally with increasing height. However, a significant decrease in the maximum load is observed when it exceeds a certain threshold (12 mm). Similar stresses are observed in out-of-plane bending tests. The maximum bending load resistance of the array decreases as protrusions are removed, and the interface area decreases. A small increase in height (from 5 mm to 8 mm) appears to be marginally beneficial to the structure; however, when it is increased significantly (to 12 mm), a notable reduction in strength is observed. Increasing the height of the overhangs, however, helps to improve the stiffness of the structure with out-of-plane loads. The superior performance of the optimized geometries can be attributed to their ability to distribute loads across multiple contact paths, reducing stress concentrations and delaying failure initiation. From the above, it can be concluded that the design of biologically inspired interlocking structural elements, through the generation of protrusions and optimization of their contact surface, is an efficient strategy to enhance their mechanical properties, in agreement with the biological concept of reference.

Biomimetic interlocking block systems present significant potential across a variety of architectural and engineering applications. Their capacity for rapid, adhesive-less assembly makes them ideal for affordable housing or temporary or demountable structures, where speed, reversibility and reduced costs and labor are important. As such, interlocking systems characterized by geometric stability and efficient energy-dissipation mechanisms could also find applications in sustainable, reusable and high-performance construction projects. Further research, however, should be conducted in order to examine how these findings can be translated into large-scale structures and to evaluate their long-term performance. From a manufacturability perspective, the proposed interlocking blocks currently demonstrate laboratory-scale feasibility and repeatability, corresponding to an early prototyping level enabled by additive manufacturing. While further work is required to assess industrial-scale production constraints, alternative manufacturing possibilities and suitable construction materials, the parametric nature of the design supports future scalability and adaptation to industrial processes.

## Figures and Tables

**Table 1:** Printer parameters.

Printer Parameter	Value
Printer Model	Ender 3 Pro
Printer Manufacturer	Shenzhen Creality 3D Technology Co., Shenzhen, China
Nozzle size	0.4 mm
Materials	PLA
Layer Height	0.28 mm
Wall Thickness	0.8 mm
Wall Line Count	2
Infill Pattern	Zig-Zag
Infill Density	50%
Outer Wall Speed	25 mm/s
Inner Wall Speed	25 mm/s
Infill Speed	50 mm/s
Printing Temperature	205 °C
Build Plate Temperature	55 °C
Cooling Fan	90%
Build Plate Adhesion	Brim
Support	Yes
Support Pattern	Lines
Support Density	10%
Print Time	3–4 h

**Table 2:** The complete set of parameters for the Galapagos evolutionary solver.

Galapagos Parameter	Value
Fitness	Maximize
Runtime Limit	Not enabled
Max. Stagnant	50
Population	100
Initial Boost	5×
Maintain	5%
Inbreeding	75%

**Table 3:** Set of design parameters of the optimized design solution of the biomimetic interlocking structural element.

Design Parameter	Value
Length	20 mm
Width	10 mm
U division	16
V division	12
Protrusion count	4
Protrusion combination	52
Protrusion height	5 mm
Main body height	15 mm
Closed BREP	Yes
Contact Area	2800 mm^2^

**Table 4:** Characteristics of the block variations created for technical evaluation of the biomimetic design concept.

Model	Protrusion Count	Protrusion Height (mm)	Contact Area (mm^2^)	Difference (%)	Volume (cc)
0	0	0	1600	-	16
1	4	5	2000	+25	16
2	8	5	2400	+50	16
3	16	5	2800	+75	16
4	16	8.5	3640	+127.5	16
5	16	12	4480	+180	16

**Table 5:** Maximum three-specimen array loads as recorded from in-plane and out-of-plane three-point flexural tests.

Model	In-Plane Load_max_ (N)	Out-of-Plane Load_max_ (N)
1	1651.85 ± 180.52	203.35 ± 10.39
2	2300.45 ± 64.56	369.55 ± 10.11
3	3526.75 ± 193.96	865.50 ± 54.59
4	3586.25 ± 38.11	996.10 ± 117.24
5	3012.70 ± 157.26	823.15 ± 86.20

## Data Availability

The original contributions presented in this study are included in the article. Further inquiries can be directed to the corresponding author.
